# Benefits of combined hind-foot alignment and medial arch reconstruction surgery in children with flexible flatfoot: a case-series analysis

**DOI:** 10.1007/s00402-025-05831-x

**Published:** 2025-04-21

**Authors:** Fabrizio De Marchi, Ilaria A. Crippa, Filippo Maria Anghilieri, Filippo Familiari, Sara Mazzantini, Garrett R. Jackson, Jorge Chahla, Lorenzo Monti

**Affiliations:** 1 Department of Orthopaedics, Foot Surgery Unit, Villa Aprica Clinical Institute, Como, Italy; 2 Department of Anaesthesiology and Critical Care, San Marco Hospital, Zingonia, Italy; 3 Department of Orthopaedics and Trauma Surgery, Magna Graecia University, V.le Europa (loc. Germaneto), 88100 Catanzaro, Italy; 4 Research Center on Musculoskeletal Health, MusculoSkeletalHealth@UMG, Magna Graecia University, Catanzaro, Italy; 5 School of Medicine, University of Milano Bicocca, Milano, Italy; 6 Rush University Medical Center, Chicago, IL USA; 7 Department of Orthopaedics IRCCS San Raffaele Hospital, Milano, Italy

**Keywords:** Pediatric, Overweight, Obesity, Deformity, Foot pain, Gait, Flexible flatfoot, Hind-foot alignment, Medial arch reconstruction

## Abstract

**Introduction:**

Although surgical alignment of the rear-foot might be sufficient to achieve acceptable correction of pronation-distortion deformity, concomitant correction of the medial arch might improve functional results.

**Methods:**

We present our experience with combined hind-foot alignment and medial arch reconstruction by in-situ naviculocuneiform arthrodesis for treatment of flexible flatfoot in children. We retrospectively evaluated clinical data available from pediatric (< 18 years old) patients treated for flexible flatfoot in our department.

**Results:**

We performed 160 surgical corrections of flat foot in 94 children over the study period. Median age was 13 (range, 12–14) years. All patients had a minimum postoperative follow-up of 24 months. Overall postoperative outcomes were optimal in 82% (n = 113/160) of cases, good in 15% (n = 24/160) of cases, and adequate in 3% (n = 3/160) of cases. At 24-month follow-up, complete surgical correction of deformity was achieved in 89% (n = 143/160) procedures. Complete consolidation of arthrodesis was achieved within 3 months form surgery in 84% (n = 134/160) of cases, between 3 and 6 months in 12% (n = 21/160) of cases. There was a significant difference in pre-operative AOFAS score among the different weight categories (p < 0.001). At post-hoc analysis, OB patients had lower AOFAS versus NW or OW patients. At 24-months follow-up, there was a significant difference in AOFAS scores among the different weight categories (p = 0.04). At post-hoc analysis, OB patients had lower AOFAS versus OW patients. There was no difference in AOFAS scores at final follow-up (p = 0.12). Postoperative pain was absent in 88% (n = 140/160) of cases.

**Conclusion:**

At a minimum 24-month follow-up, patients who undergo flat-foot deformity correction using a surgical technique combining sinus tarsi arthroeresis and medial arch reconstruction by naviculocuneiform arthrodesis experience good short-term results.

## Introduction

Pronation-distortion syndrome, also known as “flexible flatfoot” or *pes plano valgus*, is a foot deformity characterized by calcaneus-valgus weight-bearing with concomitant collapse of the plantar vault. Flatfoot is a common and typically asymptomatic pathology affecting young childhood [1, 2]. Most children develop a normal arch by the age of ten [2, 3]. However, a small percentage of children fail to develop a normal arch by adulthood [2]. Obesity in children is correlated with the tendency of the longitudinal arch to collapse in early childhood [3, 4]. The persistence of deformity in older children may lead to pain, impaired function, and fatigue with a significant impact on sports performance and quality of life. [5]. Furthermore, complications such as hallux valgus, toes deformities, posterior tibialis tendon dysfunction, and arthrosis of the peritalar joints may occur with disease progression [6, 7].

In most cases, orthotic treatment is sufficient to restore function [8]. However, surgery may be indicated in selected cases [9]. Successful hind-foot alignment can be obtained by arthroereisis using sinus tarsi implants or a calcaneus stop screw [6, 10–12], which usually achieves medial plantar arch's normalization in young children. In more complicated cases, a medialization osteotomy of the calcaneus might be necessary [10, 11], with or without Achilles tendon retraction [11]. Although an acceptable alignment of the rear-foot might be achieved by standard procedures, the vault's collapse during the stance and swing phases of the gait cycle might persist [13]. This results in inadequate gait propulsion and increases the risk of developing associated deformities. We hypothesized that concomitant correction of the medial arch might yield better functional results in older and obese patients. Several techniques have been proposed: tendon transpositions or tensioning, arthrodesis and additional osteotomies are the most frequently used [2, 14]. The in-situ arthrodesis of the naviculocuneiform (NC) joint is an option. We present our experience with combined arthroeresis and medial arch reconstruction by in-situ naviculocuneiform (NC) joint arthrodesis for the treatment of flexible flatfoot in children in a center of reference for orthopedic surgery in Northern Italy.

## Methods

### Study population and clinical evaluation

**Fig. 1:**
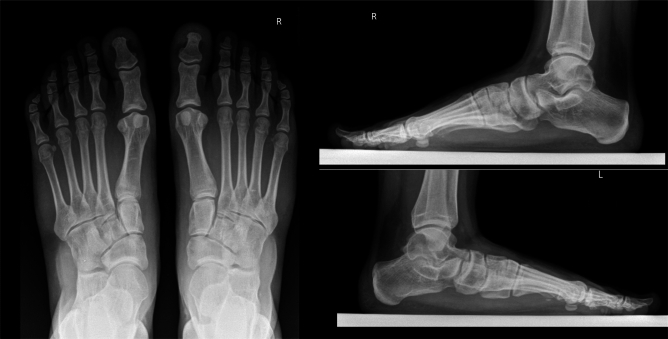
Pre-surgical weight bearing x-ray of right foot of a children affected by pronation-distortion syndrome

**Fig. 2:**
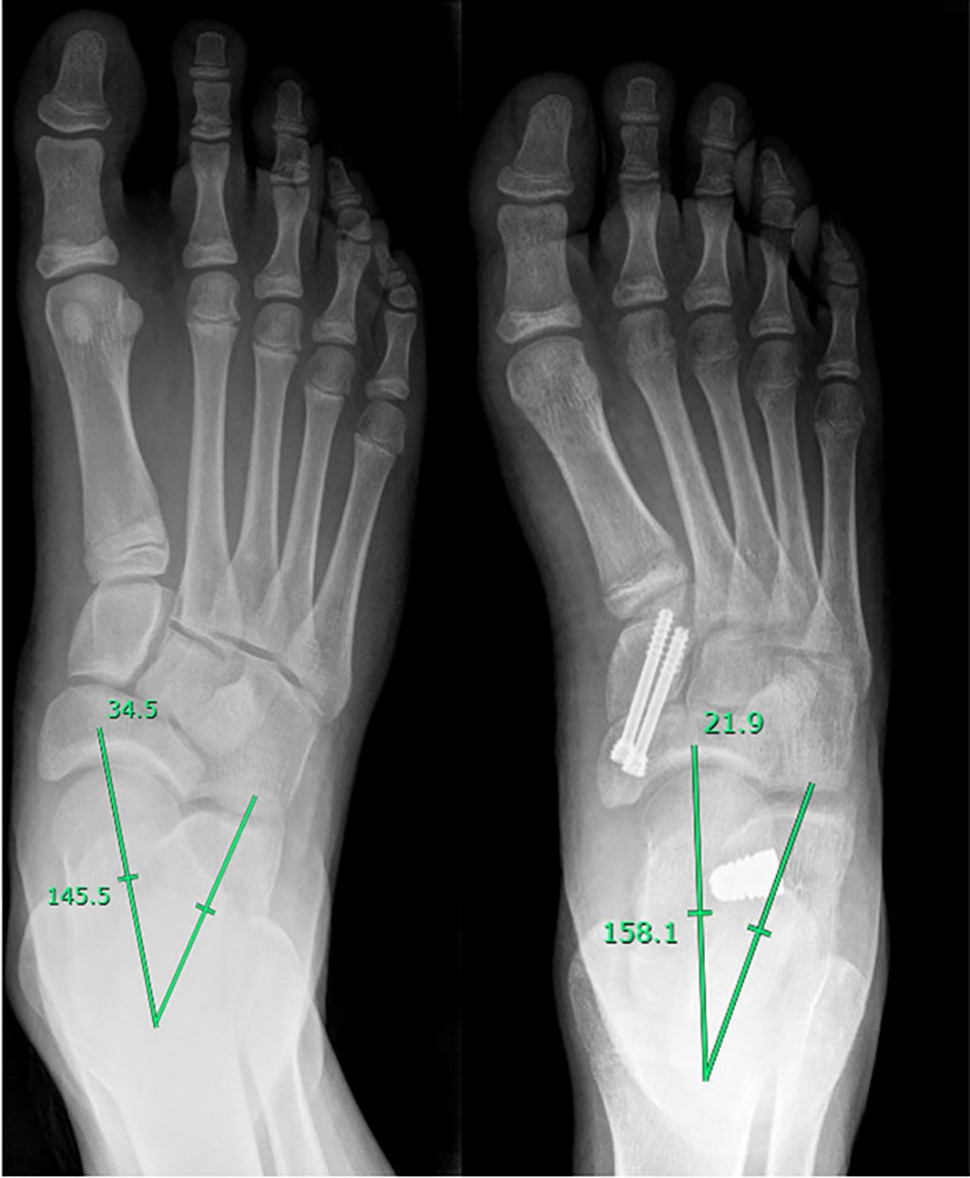
Evaluation of kite angle in a pre (left panel) and post (right panel) surgical x-ray in a left foot

**Fig. 3:**
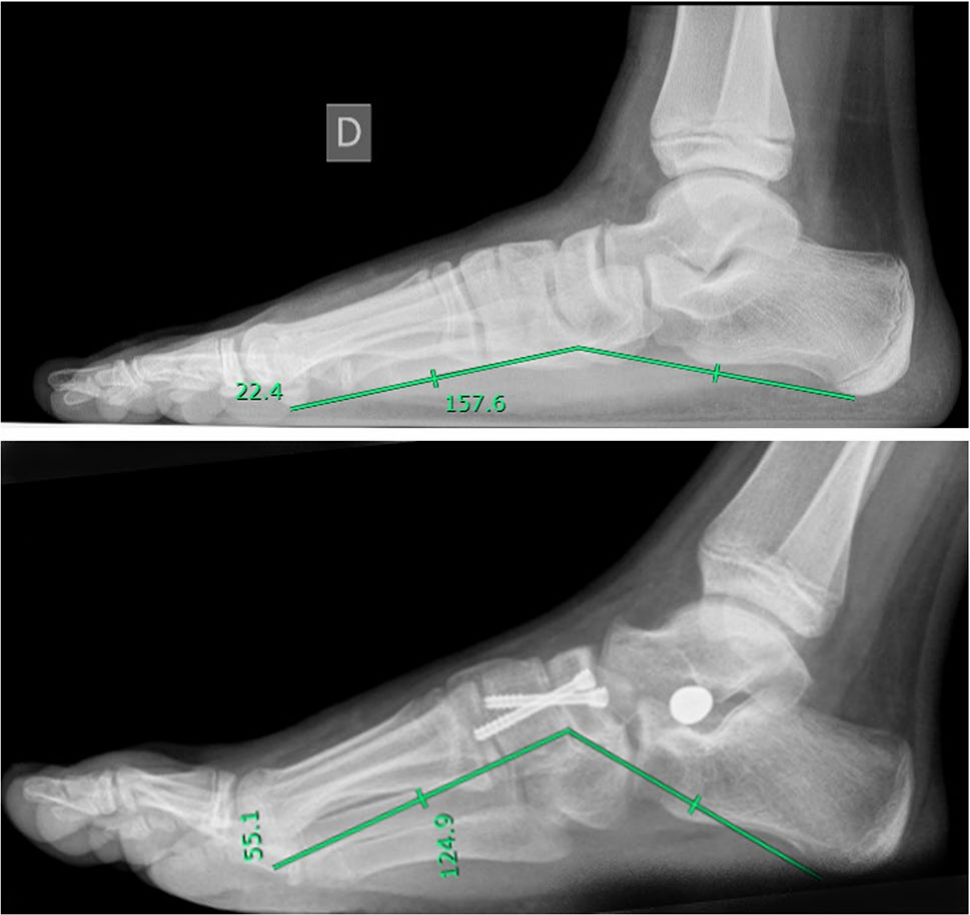
Evaluation of Costa-Bertani angle in a pre (left panel) and post (right panel) surgical x-ray in a right foot

**Fig. 4:**
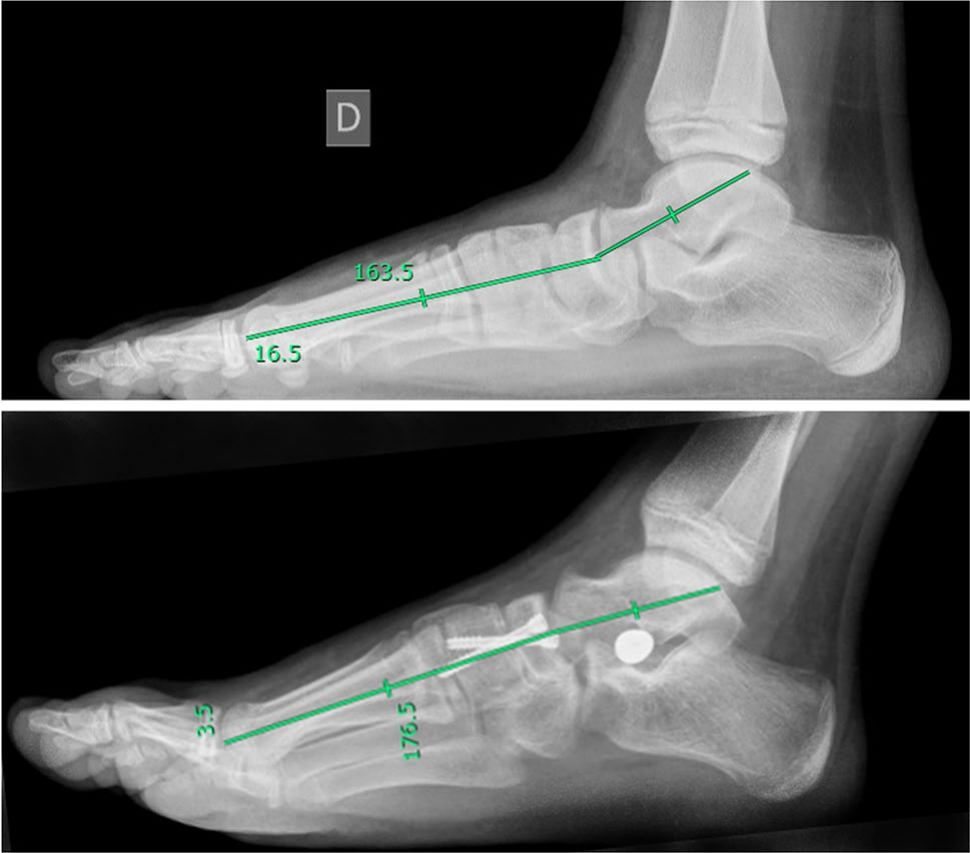
Evaluation of Meary angle in a pre (upper panel) and post (lower panel) surgical x-ray in a right foot

We retrospectively evaluated clinical data available from pediatric (< 18 years old) patients treated for flexible flatfoot from January 1^st^ 2014 to May 31^st^ 2018 in our department. Villa Aprica Clinical Institute is a hospital located in Como, Italy, specializing in the treatment of orthopedic diseases in adults and children. Children referred to our service for flexible flatfoot are treated conservatively for a minimum of 6 months or until 12 years of age, with a prescription of orthosis in presence of symptoms. Surgical treatment is considered in children > 12 years of age or in select patients ≤ 12 years who show advanced bone growth. This age cut-off was arbitrarily chosen as flat feet are still considered physiological at that age. Moreover, we arbitrarily exclude children under 12 from surgery for the fact that medial arch reconstruction through in-situ arthrodesis of the naviculocuneiform (NC) joint could bring unforeseen effects to feet with a very high growth potential yet. Indication for surgery is based upon a composite clinical and radiographic evaluation. The American Orthopedic Foot and Ankle Score (AOFAS) in its original version [7] and, from 2016 on, in its validated Italian version [15], is used to evaluate pain, functional limitations and their impact on daily activities. The minimum AOFAS Score for surgical eligibility was arbitrarily set at 75. Symptoms considered for surgical indications were pain under the medial midfoot, pain in the Achilles’ tendon area or combined knee and foot discomfort interfering with normal activities and typically occurring during or after sports practice. Without this first criterion met, no patient was considered for surgery. Additionally, the foot posture index (FPI) is usually calculated [16]. Anterior–posterior and lateral weight-bearing radiographs (Fig. 1) of the foot allows to determine the severity of foot misalignment through a set of standard parameters, such as kite angle (Fig. 2), Costa-Bertani angle (Fig. 3) and Meary angle (Fig. 4) [17].

Using these parameters, we gave indication for surgical treatment in case of Kite angle > 40°, Costa-Bertani angle > 140°, Meary angle open proximally. A single altered parameter was considered sufficient, in symptomatic patient, for surgical management; but in all cases alterations were combined.

Patients are defined as normal weight (NW) if body mass index (BMI) 18.5–24.9, overweight (OW) if BMI 25–30, obese (OB) if BMI 30.1–34.9, underweight (UW) if BMI 16.5–18.4.

Overall, we performed 160 surgical corrections of flat foot in 94 children over the study period. Characteristics of the study population are reported in Table 1. Median age was 13 (range, 12–14) years. All patients had a minimum postoperative follow-up of 24 months. Concomitant surgical procedures were performed in 34% (n = 55/160) of cases, which included the following: Achilles tendon lengthening (n = 28), syndactyly correction (n = 1); os tibialis resection (n = 4), percutaneous distal osteotomy (n = 14), screw removal (n = 3), and calcaneus-navicular synostosis resection (n = 5).

**Table 1:** Characteristics of study population

Characteristics of study population	
Age, years	13 [12–14]
Normal weight, n (%)	78 (72)
Overweight, (%)	16 (15)
Obese, (%)	13 (12)
Underweight, (%)	1 (1)
Bilateral flat foot, n (%)	70 (65)
Surgery on right foot, n (%)	76 (48)
Surgery on left foot, n (%)	84 (52)

Normal weight: BMI 18.5–24.9; overweight: BMI 25–30; Obese: BMI 30.1–34.9; underweight: BMI 16.5–18.4

### Surgical technique (Fig. 5)

**Fig. 5:**
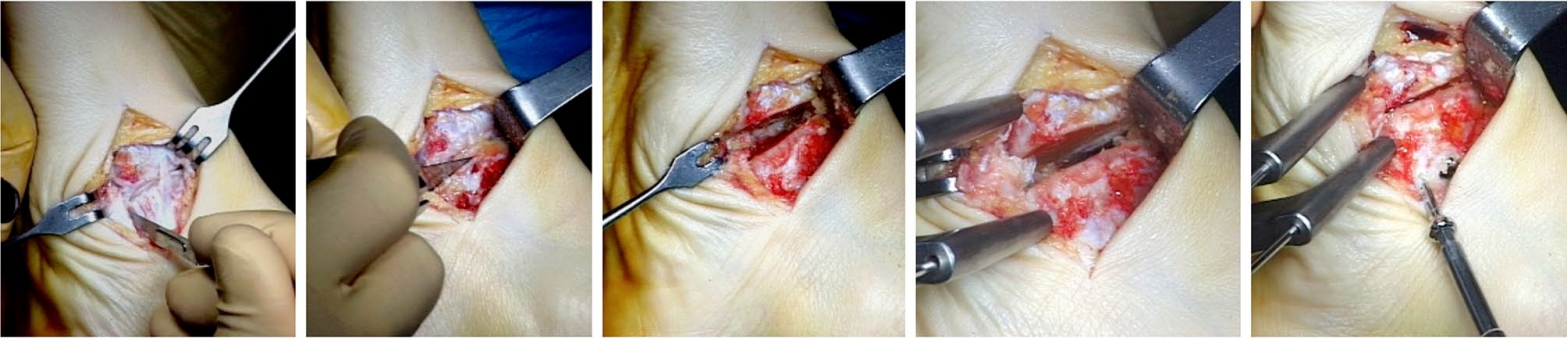
Navicular- first cuneiform joint arthrodesis surgical technique

A standard rear-foot realignment correction with seno-tarsal endorthesis was performed in all patients (sinus tarsi arthroeresis). If dorsiflexion in the neutral position was present, a percutaneous Achilles tendon lengthening was performed. In subjects approximating the end of their growth curve, a medialization calcaneal osteotomy was preferred, especially when a significant degree of rear-foot deformity was present. In addition, a surgical medial arch correction was performed. The hyaline articular surface was resected through a small (3–4 cm) medial or dorsal-medial incision. Few millimeters of the articular surfaces of the medial cuneiform and navicular bones were removed and provisional wires were inserted before two divergent double-threaded screws were placed under fluoroscopic guidance. The screws were carefully plunged to avoid the risk of protrusion. In case of advanced-stage deformities where the two bony surfaces cannot be effectively approached, a minimal resection at the base of the intermediate cuneiform was sometimes necessary. Additional surgical procedures, including prominent navicular excision, os tibiale externum, hallux valgus and other toe deformities correction, were performed as needed. Dorsoplantar (DP), medial oblique, and lateral projection radiographs were performed at the end of surgery.

### Postoperative care and follow-up

**Fig. 6:**
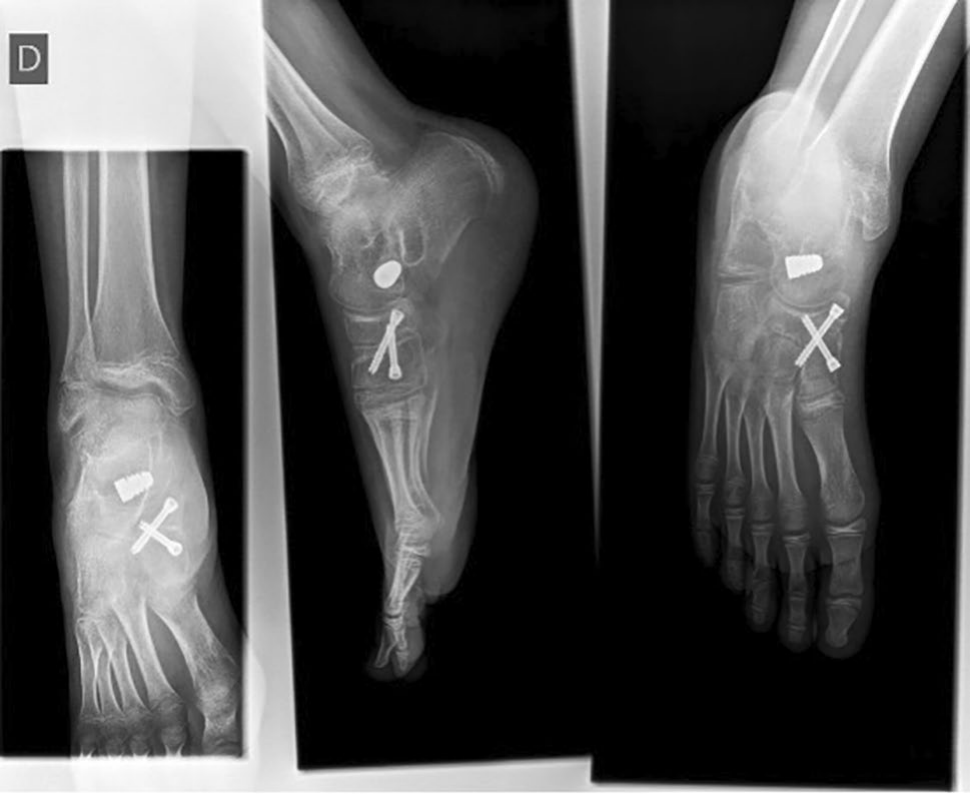
Three months post-surgical x-ray of right foot (antero-posterior, latero-lateral, oblique views)

A cast boot was maintained four weeks post-operatively. Weight-bearing was allowed as tolerated, with indication to start walking in standard footwear 7–10 days after cast removal. Gait rehabilitation therapy was suggested in children with emotional distress or difficulty due to pain or fear. Non-weight-bearing sports activities (e.g., swimming, biking) were immediately allowed. Weight-bearing activities (e.g., those implying running or jumping) were allowed 75 days post-surgery. Follow-up consultations and radiographs are performed at cast removal, 90 days, and 6 months post-operatively (Fig. 6). Follow-up consultation with plain and weight-bearing radiographs and clinical evaluation (AOFAS score calculation) was performed 24 months after surgery. In case of incomplete fusion or limited functionality, additional visits and exams are planned as needed. Hardware removal was considered in symptomatic cases. Overall result of surgical correction of flat foot was defined as optimal, good, adequate or inadequate based on clinical performance at follow-up (considering of absence of pain, walking problems, standing problems, self-consciousness about foot). Counter-lateral surgical treatment is planned 2–3 months after complete recovery, to facilitate the return to sports activities within 5–6 months.

### Statistical analysis

Statistical analysis was performed using IBM SPSS Statistics version 27 (IBM, Armonk, NY, USA). Continuous variables are expressed as median [25^th^–75^th^ percentiles]. Categorical variables are expressed as count (percent). One-way ANOVA with post-hoc Tukey test was used to test difference between weight groups. The significance level was set at p < 0.05.

## Results

53% (n = 84/160) of cases had a median follow-up of 48 [40–50] months (Table 1) 72% (n = 78/108) of patients were normal weight, 15% (n = 16/108) patients were overweight, 12% (n = 13/108) were obese, and 1% (n = 1/108) of patients were underweight. Pre-operative AOFAS was 70 (range, 66–72). Foot posture index (FPI) was 11 (range, 10–12).

### Postoperative outcomes

At the 24-month follow-up, the median AOFAS score was 100 (range, 96–100). Overall postoperative outcomes were optimal in 82% (n = 113/160) of cases, good in 15% (n = 24/160) of cases, and adequate in 3% (n = 3/160) of cases. At 24-month follow-up, complete surgical correction of deformity (defined by obtaining foot angles within physiological range) was achieved in 89% (n = 143/160) procedures; incomplete surgical correction (defined by obtaining foot angles close tophysiological range) was achieved in 10% (n = 15/160) procedures. In 0.6% (n = 1/160) of cases, the deformity was not corrected by surgical procedure and 0.6% (n = 1/160) of cases had relapse. The NC joint fusion was evaluated via foot x-rays. Complete consolidation of arthrodesis was achieved within 3 months form surgery in 84% (n = 134/160) of case, between 3 and 6 months in 12% (n = 21/160) of cases. In 0.6% (n = 1/160) of cases, consolidation was achieved at 48-months follow-up; 4% (n = 3/160) of cases did not result in proper consolidation. Of those, 2 cases required surgical revision, while other 2 showed signs of incomplete radiographic consolidation without associated symptoms or functional limitations. AOFAS score was 100 (range, 98–100) at last follow-up available (48 [range, 40–50] months, n = 84) (Table 2).

**Table 2:** Clinical and radiological evaluation before and after surgery

	Before surgery	After surgery
AOFAS score	70 [66–72]	100 [96–100]
Foot posture index	11 [10–12]	
Kite angle, °	46 [44–48]	28 [25–30]
Costa-Bertani angle, °	145 [140–150]	120 [118–125]
Meary angle, °	9 [6–12]	0 [0–2]

AOFAS American orthopedic foot and ankle society. AOFAS score was evaluated 2 years after surgery; Kite, Costa- Bertani and Meary angles were evaluated 3 months after surgery

There was a significant difference in pre-operative AOFAS score among the different weight categories (70 [range, 68- 72] in NW, 68 [range, 66–70] in OW, 65 [range, 60–68] in OB, p < 0.001). At post-hoc analysis, OB patients had lower AOFAS versus NW or OW patients. At 24-months follow-up, there was a significant difference in AOFAS scores among the different weight categories (100 [range, 96–100] in NW, 100 [range, 100–100] in OW, 96 [range, 92–100] in OB, p = 0.04). At post-hoc analysis, OB patients had lower AOFAS versus OW patients. There was no difference in AOFAS scores at final follow-up (97 [range, 100–100] in NW, 100 [range, 100–100] in OW, 99 [range, 95–100] in OB, p = 0.12).

### Postoperative complications

Postoperative pain was fully controlled with only paracetamol administration in 88% (n = 140/160) of cases. 11% (n = 18/160) of cases had fatigue-related postoperative pain requiring paracetamol and NSAIDs, while 0.6% (n = 1/160) of case has pain at rest. Boot cast had to be removed early because of intolerance in 0.6% (n = 1/160) of patients. In 2 cases, sign of superficial infection required a short cycle of antibiotic therapy and regular wound dressing to allow second-intention closure. Arthrodesis failed to consolidate in 1% (n = 2/160) of cases and required surgical revision. Achilles tendon repair was needed in one patient due to trauma. Calcaneo-stop screw removal was necessary in 6% (n = 9/160) of cases because of intolerance. In one patient, the intolerance to “calcaneo-stop” screw was likely due to peroneal tendonitis, not related to the medial arthrodesis. Gait rehabilitation therapy was prescribed by the specialty doctor or required by patients’ guardians in 16% (n = 25/160) of cases. An orthotic insole was prescribed in 4 cases: for 1.2% (n = 2/160) of cases of persistence of late-consolidation pain and for other 1.2% (n = 2/160) of cases of weight-bearing, competitive sport practice (track and field).

## Discussion

Patients underwent a novel flat-foot deformity surgical correction technique that combines traditional hind-foot alignment and medial arch reconstruction which resulted in excellent patient outcomes among normal weight and obese children at a minimum 24-month follow-up. In particular, excellent clinical and radiological outcomes could be obtained through a simple minimally-invasive arthrodesis of the naviculocuneiform (NC) joint in adolescents with structural bone deformity and limited growth perspective, independently from BMI.

*Pes plano valgus* is a complex deformity, characterized by plantar flexion and eversion of the calcaneus against the tibia, plantar flexion of the talus, and forefoot varus. It is frequently associated with instability and collapse of the plantar vault. The deformity is often asymptomatic as long as the foot structures are flexible, like during childhood [8]. However, as soon as skeletal ossification progresses during adolescence, the mid and forefoot adapt to the hind-foot deformity, leading to progressive over-supination. Over time, such adaptive mechanism determines permanent deformity and associated compensatory foot deformities might ensue, e.g., hallux valgus, hallux limitus, and toes’ alterations. Associated proximal lower-limb joint disorders might also develop later during adulthood [18]. There is a general consensus that children over 12 years with marked deformity, pain and impaired function might be eligible for surgical correction to halt the progression of the condition [19]. However, there is no consensus on the best surgical technique. The"calcaneo-stop” procedure is very commonly performed. It consists of arthroeresisof the subtalar joint in order to obtain talocalcaneal alignment. Several authors support the efficacy of this procedure, particularly in younger populations (10–12 years old). However, in a systematic review on 756 feet, Metcalfe et al. reported a frequency of patient satisfaction between 79 and 100%, a relevant frequency of surgical complications (from 7.1% to 19%) [20]. Common reported post-operative issues were painful tarsal sinus, hardware extrusion, and unsuccessful correction of the deformity [21]. Hind-foot alignment surgery alone, although well performed, may result in satisfactory bipodalic support static configuration, but under-perform during gait. The calcaneal realignment might not be sufficient to correct an advanced hind-foot varus deformity with evidence of a collapsing arch at the navicolocuneiform joint level. In such cases, during the gait stance phase, initial contact occurs with a correct heel strike. However, during the flat and late-midstance phase, until just the beginning of the push-off phase, a progressive premature shift towards medial weight distribution occurs. Medial arch collapse and less effective propulsion are then inevitable consequences. Medial arch reconstruction surgery may help achieve correct biomechanics during gait. Several surgical techniques have been proposed, each with advantages and disadvantages [22]. The technique we describe aims to guarantee a plantar stance without altering tendinous structures. A minimal reduction in talo-navicular joint motion range is a theoretical risk without actual development: advanced foot deformity is per se associated with reduced talonavicular mobility, which is usually not further reduced by the procedure. Another advantage of the procedure is that it does not require prolonged immobilization or hardware removal. The surgical technique we perform can be considered a variation of the Cotton osteotomy technique, in which a dorsal base bone wedge is inserted at the first cuneiform level [23]. However, our modified technique has several important advantages compared to the traditional Cotton technique: proper consolidation is achieved in shorter time compared to Cotton technique, thus reducing outcome uncertainty relative to variations in bone reabsorption or wedge protrusion; the stability of the fusion and the avoidance of grafts or bone wedges, allow for the early tolerance of weight-bearing. Furthermore, such technique carries a low risk of long-term relapses, which is a constant possibility in tendon transposition techniques. Nevertheless, a successful hind-foot alignment surgery is a prerequisite and can be performed with several different techniques. We perform an extra-articular subtalar arthroereisis in patients with some residual growth perspective, so to preserve a complete subtalar mobility. In patients with advance bone growth and advanced deformity, a medialization calcaneal extra-articular osteotomy is preferred.

The combined technique of hind-foot alignment and medial arch reconstruction we presented was able to achieve overall optimal clinical and radiological result in the vast majority of patients. The incidence of complications directly attributable to the NC joint fusion was minimal. Specific attention was reserved for the risk of delayed consolidation or pseudoarthrosis. The incidence of pseudo-arthrosis in our cohort was limited compared to previous studies [24]. The use of a bone-sparing technique with careful limitation of the articular surface removal and the approach of the two bones without interposed grafts might have been a key factor. We are aware that the angular deviation might have been left untreated by not removing bone wedges. However, in our population, a correct alignment could be achieved anyway, as confirmed by imaging. We believe this to be at least partially related to how a proper hind-foot correction procedure might itself benefit the internal arch alignment, thereby leading to a more favorable working axis for the subtalar joint. Several authors have reported this same effect, both with arthrodesis [20] and calcaneal osteotomies [25]. Failed corrections or relapses of flatfoot in patients with advanced forefoot deformities might be based on the medial column's instability [26]. In such cases, even with a successful hind-foot alignment correction, with bearing a progressively increasing weight over time, the medial arch gradually collapses, thereby resulting in a persistent forefoot varus. The talonavicular, or cuneometatarsal arthrodesis targets such instability, but with the disadvantage of a global stiffening of the ankle. The additional medial arch reinforcement we performed in our cohort does not affect ankle mobility and might be a key contributor to reduce relapses.

### Limitations

Our study has limitations. We retrospectively reported a single-center experience with a novel surgical technique. Therefore, selection bias cannot be excluded and inference of any definite conclusions on the efficacy of the technique needs further validation. Furthermore, it’s important to note that the short-term follow-up in children may not reveal effects that may develop years later, such as early osteoarthritis of contiguous joints. However, we were able to present data on a large cohort of patients with a significant number of surgical procedures performed, thus laying solid foundation for further investigations.

## Conclusion

At a minimum 24-month follow-up, patients who undergo flat-foot deformity correction using a surgical technique combining sinus tarsi arthroeresis and medial arch reconstruction by naviculocuneiform arthrodesis experience good short-term results.

## Data Availability

Institute Villa Aprica anonymized database is used for data collection and storage.
